# Fracture Initiation in Notched Specimens Subjected to Compression: Strain Rate Effect

**DOI:** 10.3390/ma13112613

**Published:** 2020-06-08

**Authors:** Elżbieta Bura, Andrzej Seweryn

**Affiliations:** Faculty of Mechanical Engineering, Bialystok University of Technology, Wiejska 45C, 15–351 Bialystok, Poland; a.seweryn@pb.edu.pl

**Keywords:** fracture, polymeric material, viscoelastic material, notches, strain rate effect

## Abstract

This paper shows the results of an experimental investigation on fracture in polymethyl methacrylate (PMMA) notched specimens subjected to compression (with unloading) including different strain rates. Three types of notches were used. Flat specimens were weakened by two types of V-notches and U-notches. Additionally, two specimen thicknesses were used (9.7 and 14.5 mm). The load was carried out at the strain rate of 8 × 10^−4^, 4 × 10^−3^, and 2 × 10^−2^ s^−1^ and the unloading stage was conducted ten times faster, i.e., 8 × 10^−3^, 4 × 10^−2^, and 2 × 10^−1^ s^−1^, respectively. By using a PHANTOM high-speed camera, fracture initiation moments and locations were indicated. Two types of crack were observed and distinguished as A-type and B-type. The first was formed by the contact stress of the closing notch surfaces, while the latter was formed by the residual stresses during the unloading stage. The type of notch, specimen thickness, and the strain rate have a significant influence on the fracture process. The strain rate has a large impact on the critical load value, which determines the fracture initiation, but does not affect the location and shape of the crack. The strain rate effect usually disappears with increasing specimen thickness.

## 1. Introduction

Low density and low production cost make thermoplastics such as polymethyl methacrylate (PMMA), more and more widely used in industry [1,2,3]. The great popularity of plastics requires testing them in various conditions. Much of this research has focused on observing the fracture phenomenon that elements weakened by notches are particularly susceptible to. The tests have taken into account the influence of various factors on the fracture process, such as notch type, loading conditions, specimens shape, etc. The fracture phenomenon has been described for axisymmetric specimens during mode III and mixed-mode I/III [4,5], as well as discussed for many types of plane specimens used for compression, tensile, or shear tests. The fracture phenomenon has been analyzed in polymers weakened by sharp-edged V-notches [6], central key-hole notches [7], central rhombus-shaped notches [8], or semicircular notches [9,10,11]. In the above-mentioned works, the authors aimed to formulate the fracture criterion, which took different forms depending on the type of specimen and its exploitation conditions. Experimental research is the basis for theoretical predictions but the testing and description of this process in glassy polymers is difficult, mainly due to their high sensitivity to temperature and strain rate changes.

Flat PMMA specimens have been tested in a tensile test using different strain rates (2.92 × 10^−5^, 2.38 × 10^−4^, 2.01 × 10^−3^, and 2 × 10^−2^ s^−1^) [12]. As the strain rate increased, the Young’s modulus and tensile strength increased. The material characteristics changed from strongly nonlinear to linear, and the higher the strain rate, the lower the material softening. The fracture surfaces were analyzed using a SEM scanning microscope. As the strain rate increased, a reduction in the perfectly smooth area was observed (the place of fracture initiation) and the majority of the fracture surface was irregular. Torsion tests at different strain rates (500–2200 s^−1^) and temperature in the range of −100–200 °C were carried out on PMMA and PC specimens [13]. The undoubted influence of the deformation rate on the character of the torsion curve was found. Different sensitivities to changes in deformation rate were observed during tests at room temperature, which was related to different relaxation times.

Amorphous polymers including PMMA have more complex characteristics in uniaxial compression than tension or torsion. The concept of viscosity becomes more visible for this load type. Assuming that PMMA represents viscoelastic materials, the following stages should be distinguished during the compression process: viscoelastic (up to the yield point), strain softening/yield drop [14], and then strain hardening [15], leading to destruction [16]. The occurrence of these phenomena makes it difficult to analyze and describe the behavior of amorphous polymers during compression. One of the first studies concerning the fracture phenomenen during compression was described in [17]. Dynamic (strain rate in a range of 10^−4^–10^3^ s^−1^) compression tests of several popular polymers such as PMMA, PC, PBT, or PVD were performed. For all polymers, a higher yield point was observed, the higher the deformation speed was set. The same compression strain rates were used as in the investigation described by [18]. Once again, the strong influence of strain rate on PMMA and PC characteristics was confirmed. PMMA showed the characteristics of linearly elastic material at the highest strain rate, however, during the destruction of the material (plastic deformation) heating was observed, which excluded its ideal brittleness. Studies on the influence of strain rate and temperature on the mechanical properties of PMMA during compression, using axisymmetric specimens were presented in [19]. Three amorphous polymers, i.e., polycarbonate, polymethyl methacrylate, and polyamideimide were subjected to compression tests at various ambient temperatures (−40–180 °C) and at various strain rates (0.0001–5000 s^−1^) [20]. The results showed that Young’s modulus, yield strength, and hardening rate were strongly dependent on the strain rate. As the strain rate increased, the value of all these quantities increased, and the same was reported for all tested polymers. A study by [21] described tensile fracture tests using contact-tension (CT) specimens made of isostatic polypropylene and its modified version. The components were tested at different speeds of 0.1 mm/s–14 m/s. The differences in the course of fracture processes were indicated for both materials. The homopolymer showed a change of character from ductile to brittle with an increase in the test speed (craze), while the modified material was characterized by a stable crack propagation over the entire speed range. By using specimens of different thicknesses, it was found that the increase in the size of the crack tip damage zone, together with the increase in the load application speed, was related to the change of the plane stress to plane strain state.

The main purpose of fracture research is to formulate criteria to predict the process that prevents its negative effects, i.e., failure of elements or entire structures, which is possible only by getting to know a fracture in advance based on experimental research. Studies on fracture during compression of notched polymers have been presented in many papers. Tested elements have been weakened by rhombus-shaped central notches [22], square rounded corners hole [23], central circular hole [24], key-hole notch [7], rounded V-notch [25], and many others. Each paper presents an experimental procedure leading to the determination of the fracture criterion for a given specimen type during compression. However, none of these works took into account different strain rates. The influence of this magnitude on the fracture process during compression in amorphous polymers with stress and strain concentrators was not determined. Most of the existing fracture criteria are used for notched polymer specimens, subjected to only those states in which the material shows constant linearly elastic behavior. It is important to supplement these tools with a criterion which can predict fracture processes in polymers that show behaviors other than the typical brittle behavior, for example, viscoelastic. This can be achieved by taking into account various parameters such as a strain rate or temperature during experimental tests. Such an approach results in a full picture of the fracture process, leading to the formulation of new tools for predicting fracture.

This paper describes the course of experimental fracture tests carried out on flat specimens made of polymethyl methacrylate (PMMA). The specimens were weakened with V-type edge notches with opening angle ≈ 90° (with the root radii close to *R* ≈ 0 mm and *R* ≈ 1 mm) and U-notches (*R* ≈ 10 mm). Uniaxial monotonic compression tests with the unloading stage, were carried out. The tests were carried out at three different strain rates (8 × 10^−4^, 4 × 10^−3^, and 2 × 10^−2^ s^−1^). Two types of crack were observed, one during the loading stage and the other during the unloading stage. A PHANTOM camera was used to determine the moments of their initiation and locations. The effect of strain rate and specimen thickness on the course of the fracture process was described. The higher the strain rate, the higher the compressive force causing the initiation of fracture. However, the strain rate did not significantly affect the location and shape of cracks. Its influence on the fracture process was noticed only at a strain rate higher than 4 × 10^−3^ s^−1^. This rate effect disappeared as the thickness of the specimen increased. This research provides a detailed overview of the fracture phenomenon of PMMA during compression with unloading and the results provide the basis for formulating fracture criteria taking into account different strain rates.

## 2. Specimens

**Figure 1:**
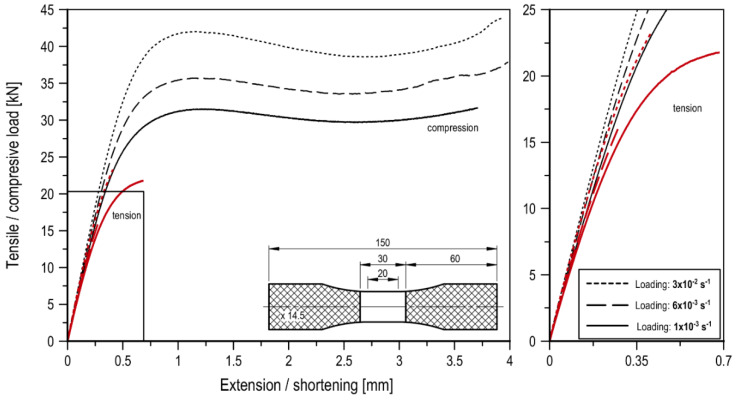
Tension and compression curves for the smooth specimens at different strain rates (tension, red line and compression, black line).

Polymethyl metacrylate, which is a thermoplastic polymer with very good optical properties, was the subject of this research. The basic material properties were determined as a result of tensile and compression tests performed on smooth specimens (without notches). The data for loading speed equal to 0.02 mm/s (strain rate equal to about 1 × 10^−3^ s^−1^) are presented in Table A1 [26]. Additionally, the smooth specimens with the measuring base equal to 15.7 ± 5 mm, were also subjected to tensile and compression tests at different strain rates, i.e., $\dot{\varepsilon }$ 1 × 10^−3^, 6 × 10^−3^, and 3 × 10^−2^ s^−1^. The force elongation/shortening curves are shown in Figure 1. In order to avoid buckling of the specimen, the height of the specimen (between the handles) did not differ significantly from the other dimensions. The surfaces of the specimen that were placed in the jaws were hatched (Figure 1).

**Figure 2:**
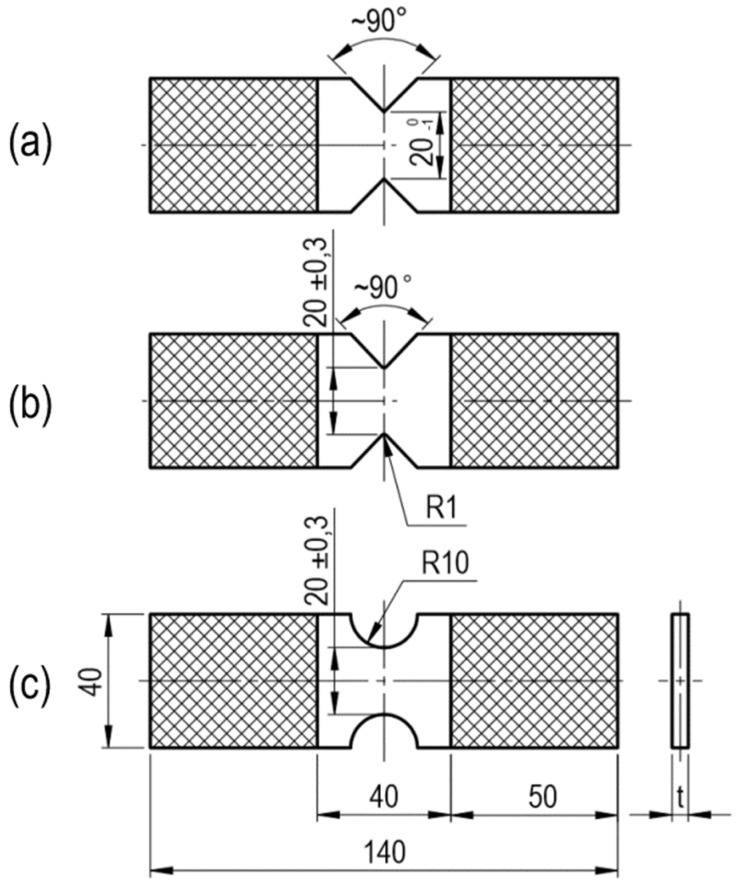
The V-notched specimen with (**a**) a root radius equal to *R* ≈ 0 mm; (**b**) a root radius equal to *R* ≈ 0 mm; and (**c**) the U-notched specimen (*t*, specimen thickness equal to 9.7 and 14.5 mm).

Flat specimens with a thickness of *t*_1_ = 9.7 mm and *t*_2_ = 14.5 mm were subjected to monotonic compression tests (with unloading). The components were weakened with the following edge notches: V-type with a root radius equal to *R* ≈ 0 mm (sharp V-notch) and an opening angle equal to 90°, V-type with a root radius equal to *R* ≈ 1 mm and an opening angle equal to 90° (rounded V-notch), and also U-type with a root radius equal to *R* ≈ 10 mm. The specimens were placed in the testing machine jaws so that the measuring area was 40 mm. The shape and dimensions of the specimens are shown in Figure 2. The hatched area corresponds to the specimen volume in the testing machine jaws. The specimens’ dimensions are summarized in Table A2, Table A3 and Table A4.

## 3. Station for the Experimental Tests

**Figure 3:**
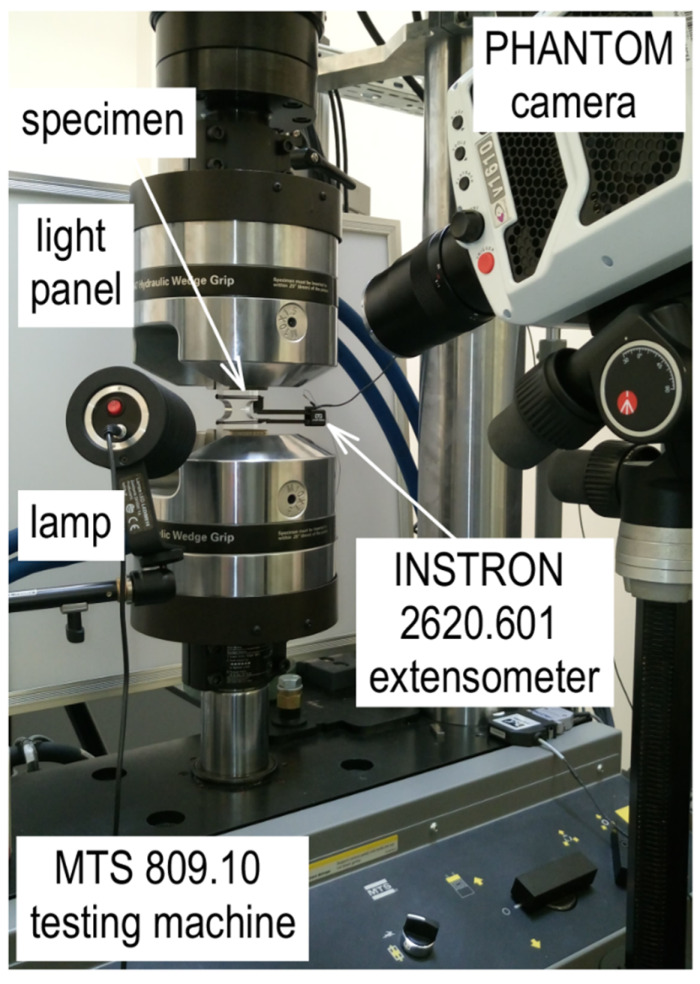
Testing fixture.

Monotonic compression tests with unloading, at an ambient temperature (20 °C) were carried out on a test stand, as shown in Figure 3. The MTS 809.10 biaxial dynamic testing machine with a hydraulic drive was used. The specimen was placed in knurled jaws and controlled by an INSTRON 2620.601 extensometer with a 25 ± 7 mm measuring base. Displacement control tests were carried out at the following measuring base shortening rates: 0.02 mm/s, 0.1 mm/s, and 0.5 mm/s, corresponding to the mean values of strain rates 8 × 10^−4^, 4 × 10^−3^, and 2 × 10^−2^ s^−1^. The load was released (force channel) ten times faster, i.e., ${\dot{\varepsilon }}_{2}$ of 8 × 10^−3^, 4 × 10^−2^, and 2 × 10^−1^ s^−1^, respectively. Three different strain rates were used to examine their influence on the fracture process, among other things. Additionally, an important parameter was the level of the maximum measuring base shortening, *u*_max_, from which the unloading process was started. Three levels of maximum displacement, i.e., *u*_max_ = (93, 5, and 7 mm) were adopted. A total of 108 tests were carried out. All the tests were monitored using a PHANTOM v1610 monochromatic camera, which took pictures at very high speeds. The purpose of the recordings was to capture the fracture initiation locations and moments and to provide a complete picture of the deformation process. In order to make maximum use of the camera’s capabilities, different recording rates were used, which depended on the duration of the entire process. The parameters are listed in Table 1. Recording time was closely related to the recording rate. In order to synchronize the results from the camera with the MTS testing machine, the coupled devices started recording simultaneously. This required differentiation of the recording speed. In the longest process, a rate of 2000 frames/s could only be used, whereas in the shortest process, using the same speed would not indicate the moment of fracture initiation. It was assumed that the recording rate was increased as the loading rate was increased accordingly. The use of the PHANTOM camera required lighting of the working space. For this purpose, the test stand was equipped with a light panel and spot lamp.

## 4. Results

**Figure 4:**
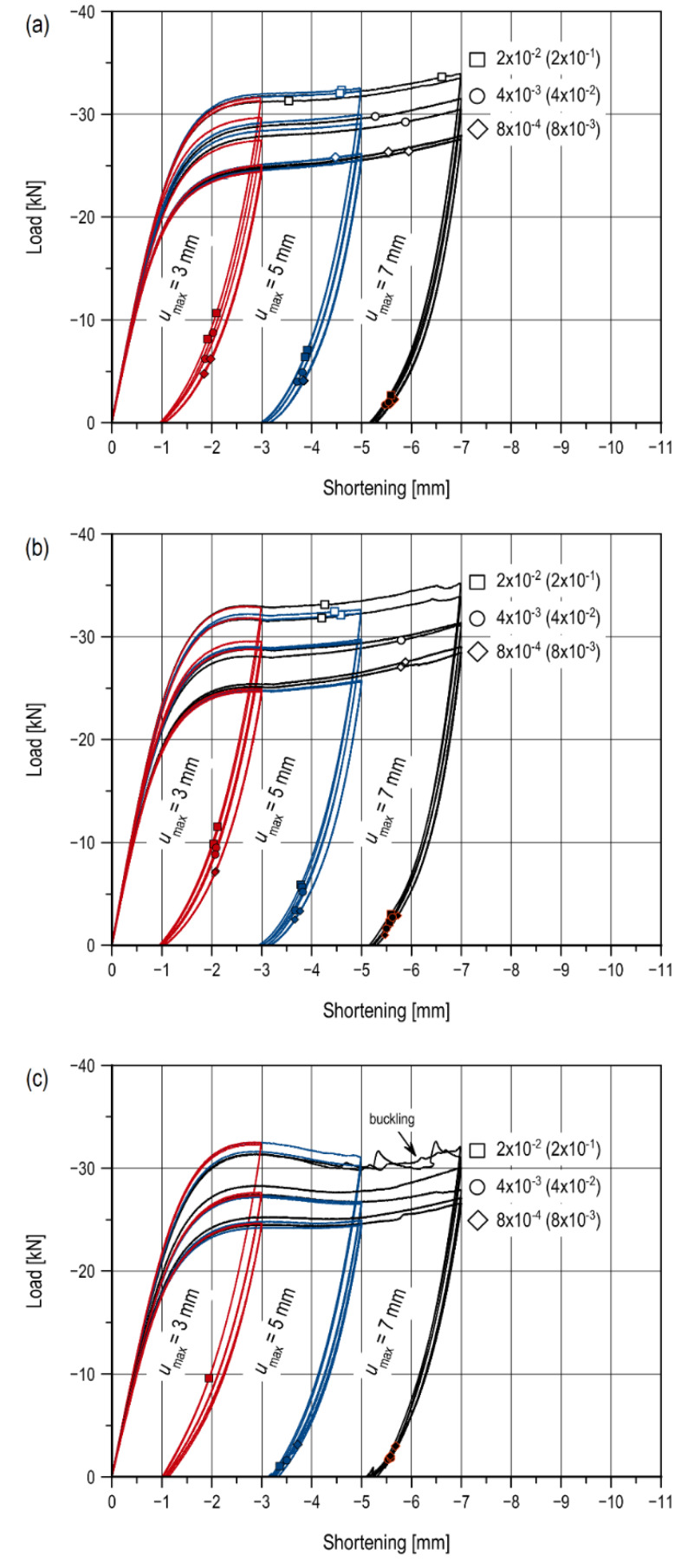
Compression curves with fracture initiation points for V-notched specimens with (**a**) root radius *R* ≈ 0 mm; (**b**) root radius *R* ≈ 1 mm; and (**c**) for U-notched specimens (*t*_1_ = 9.7 mm).

**Figure A1:**
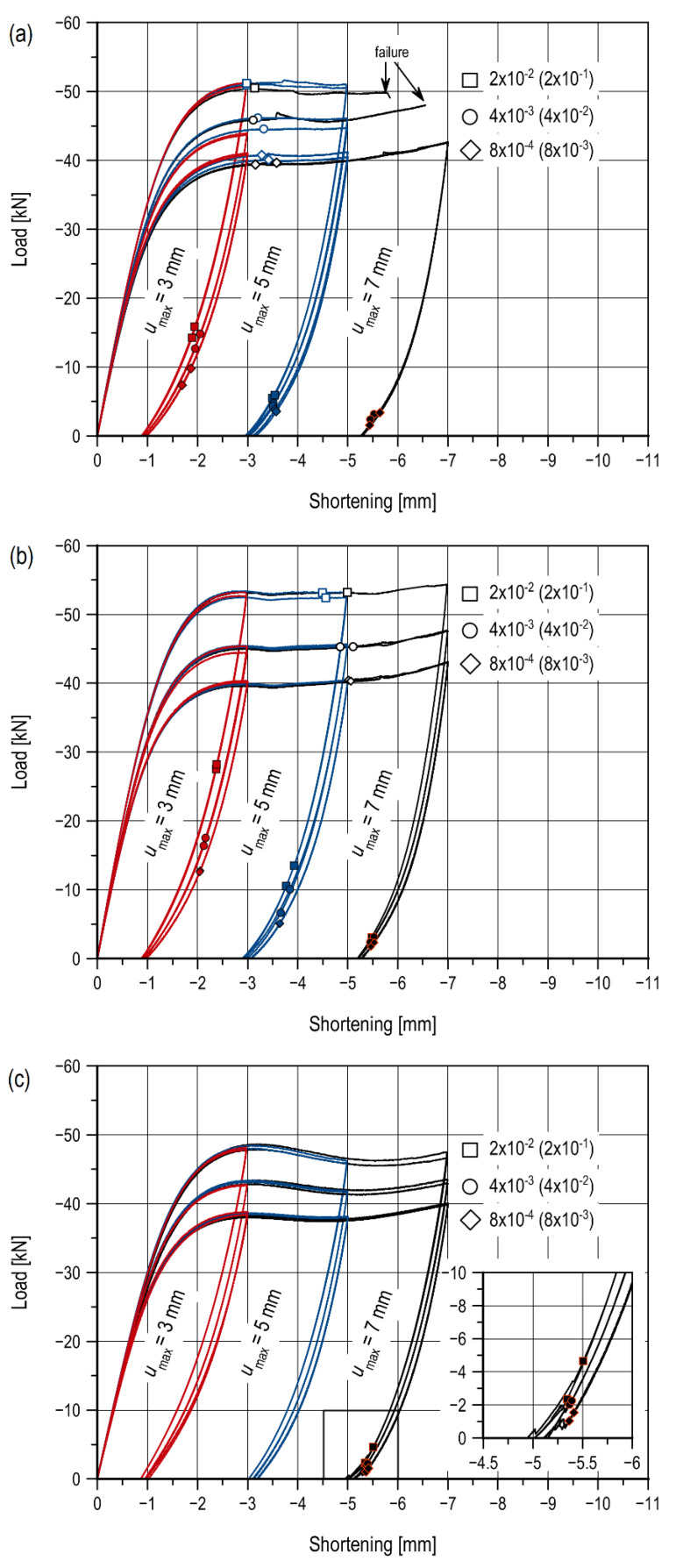
Compression curves with fracture initiation points for the V-notched specimens with (**a**) a root radius *R* ≈ 0 mm; (**b**) root radius *R* ≈ 1 mm; and (**c**) for the U-notched specimens (*t*_2_ = 14.5 mm).

As a result of the tests, the force curves representing the shortening of the measuring base for all types of notches and specimen thicknesses were obtained. Figure 4 presents data for specimen thickness *t*_1_. The different colors correspond to the successive maximum levels of the measuring base shortening (red, 3 mm; blue, 5 mm; and black, 7 mm). The points marked on the curves refer to the moment when the first crack occurs, i.e., during loading (blank points) and during the unloading stage (filled points). The same curves for specimen thickness *t*_2_ are presented in Figure A1. The figures are summarized in Table A2 and Table A3. The points were collected during the analysis of recordings from the PHANTOM camera. The moment of fracture initiation was taken as a frame preceding the one in which the crack was visible for the first time. Thanks to the synchronization of the camera and the testing machine, each frame from the recording corresponded to a specific indication of the machine.

**Figure 5:**
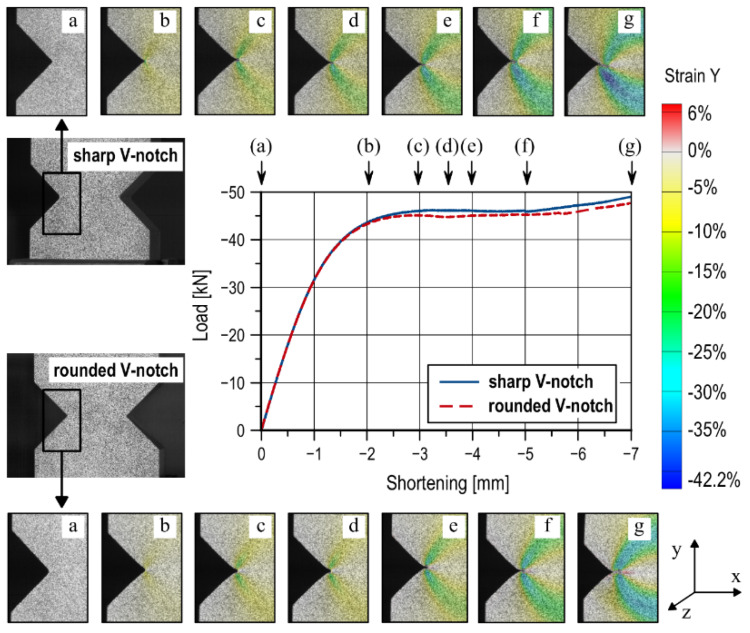
The process of notches closing in specimens of thickness *t*_2_ at a strain rate of 4 × 10^−3^ s^−1^.

For V-notched specimens, an increase in the value of the maximum compressive force is noted together with an increase in the measuring base shortening *u*. For sharp V-notched specimens, this increment is continuous (Figure 4a and Figure A1a), whereas the curves for rounded V-notched specimens (Figure 4b and Figure A1b) are characterized by a local decrease in load value at a base shortening level equal to *u* ≈ 3.5 mm (*t*_1_) and *u* ≈ 3 mm (*t*_2_). This fault is the result of the notch closing. In the case of sharp notches, the closing process begins with the application of the load. In specimens with V-notches with *R* ≈ 1 mm, contact between the notch surfaces takes place later, which is explained by the rounded bottom. A comparison of the process of V-shaped notches with different root radii is shown in Figure 5. The diagram shows the level of notch surface closure in specimens of thickness *t*_2_ at a strain rate of 4 × 10^−3^ s^−1^. A closer wall of the specimen was observed with the ARAMIS 3D 4M optical system. In addition, the distribution of strain Y component (on the load direction) is shown. Views of one of the notches were set for the following selected levels of measuring base shortening: 0, 2, 3, 3.5, 4, 5, and 7 mm. The beginning of the notch closure process, in the case of specimens with a rounded notch, was obtained later than in the case of a sharp notch. An evident notch closure (at the free surface) is noted at a shortening equal to 5 mm (Figure 5, “f” stage), whereas, for sharp notches, it is noted at a shortening equal to 3 mm (Figure 5, “c” stage). Rounding at the bottom of the notch locally delays the contact of the closing surfaces to bring them together at a critical point.

The increase in the compressive force value is due to the increased contact area and, for thicker specimens, the critical contact area is reached faster. The compression curves for U-notched specimens present different characteristics (Figure 4c). In this case, a decrease in the value of the compressive force is strongly visible, followed by an increase until the end of the loading stage. This is due to the material softening, which is subject to load when the yield point is exceeded. Unlike V-notched specimens, in this case no contact is observed. The character is similar to the compression curves of smooth specimens (Figure 1), in which the following three stages can be distinguished: viscoelastic deformation (up to the yield point), softening (decrease in the compressive force value), and hardening (finale load increase).

To eliminate dimensional errors of specimens that occurred during the production stage, the values of the effective load *F*_eff_ (i.e., the real maximum compressive force, *F*_max_, multiplied by the correction factor *ξ*) were analyzed. Where, *ξ,* is the ratio of the nominal cross-sectional area of the specimen *A*_nom_ measured at the bottom of the notch to its actual *A*_real_ dimensions (*F*_eff_ = *ξ* · *F*_max_, where *ξ* = *A*_nom_/*A*_real_).

For specimens with a nominal thickness of *t*_2_, an increase in the effective load, *F*_eff_, is observed regardless of the applied strain rate, $\dot{\varepsilon }$, and the notch type. In the V-type sharp notched specimens, for all rates, the *F*_eff_ in the displacement range *u* = 3–5 mm increased by 0.3–3.79%, and for *u* = 5–7 mm it increased by 0.66–4.80%. In specimens with rounded V-notches, for all rates, the *F*_eff_ in the base shortening range *u* = 3–5 mm increased by 0.06–1.13%, whereas for *u* = 5–7 mm it increased by 2.44–5.99%. The higher the strain rate $\dot{\varepsilon }$, the smaller the increase. For sharp notches (*R* ≈ 0 mm) as compared with rounded V-notches (*R* ≈ 1 mm), there is a greater effect of rate $\dot{\varepsilon }$ on the increase in effective load *F*_eff_ at lower levels of base shortening *u*. In the case of rounded notches, greater differences resulting from the change in the strain rate $\dot{\varepsilon }$ are observed at higher levels of the measuring base shortening *u*. U-notched specimens show different trends. For all rates $\dot{\varepsilon }$, the effective load *F*_eff_, in the displacement range *u* = 3–5 mm, increased by 0.05–0.95%, and for u = 5–7 mm it increased by 0.57–5.71%. The highest increase was observed for a rate of $\dot{\varepsilon }$ = 2 × 10^−2^ s^−1^ and the lowest increase was observed for $\dot{\varepsilon }$ = 4 × 10^−3^ s^−1^. Thus, unlike in the case of the V-notched specimens, in this case, the effective load increments are not proportional to the strain rate $\dot{\varepsilon }$.

Similar trends, but a faster increase in effective load value with increased strain rates, were observed in thinner specimens (*t*_1_). In sharp V-notched specimens, for all rates, the *F*_eff_ in the displacement range *u* = 3–5 mm increases by 1.66–4.19%, whereas for *u* = 5–7 mm it increases by 4.62–7.91%. For specimens weakened by rounded V-notches, these increases are 1.31–4.45% for *u* = 3–5 mm and 5.55–12.67% for *u* = 5–7 mm. Thus, reducing specimen thickness results in a doubling of the effective load *F*_eff_ percentage increase at each loading step. At the same time, for the maximum measuring base shortening level, *u*_max_ = 7 mm, the effect of specimen thickness disappears as the rate $\dot{\varepsilon }$ decreases, and the notch root radius increases. The effective load values for *u*_max_ = 7 mm increase for thicker specimens as compared with thinner specimens by an average of 0.58%, 2.21%, 5.30% (sharp V-notches), 2.48%, -%, 3.64% (rounded V-notches), respectively, for the strain rate $\dot{\varepsilon }$ equal to 8 × 10^−4^, 4 × 10^−3^, and 2 × 10^−2^ s^−1^. As for thickness *t*_2_, thinner specimens weakened by U-notches are subjected to different dependences of the increase in the effective load *F*_eff_ value with the change in rate $\dot{\varepsilon }$ than those with V-notches. Changes in the strain rate $\dot{\varepsilon }$ affect the increase in the *F*_eff_ by 0.75–5.22% for *u* = 3–5 mm, and 4.79–8.82% for *u* = 5–7 mm. With an increase of rate $\dot{\varepsilon }$, the increase of effective load value *F*_eff_ for *u* = 3–5 mm is greater, whereas for *u* = 5–7 mm the increase is less. For all notch types and levels of the measuring base shortening *u*, the same permanent base shortening is observed (at the end of the unloading), regardless of the applied strain rate $\dot{\varepsilon }$. Two types of crack were observed during the tests, similar to the tests shown in [26]. During loading, A-type cracks in the shape of the dragonfly wings appeared, while during the unloading stage there were B-type cracks which initiated at the notch root. The causes and moments of their occurrence are thoroughly discussed in the following sections.

### 4.1. Fracture During Loading

**Figure 6:**
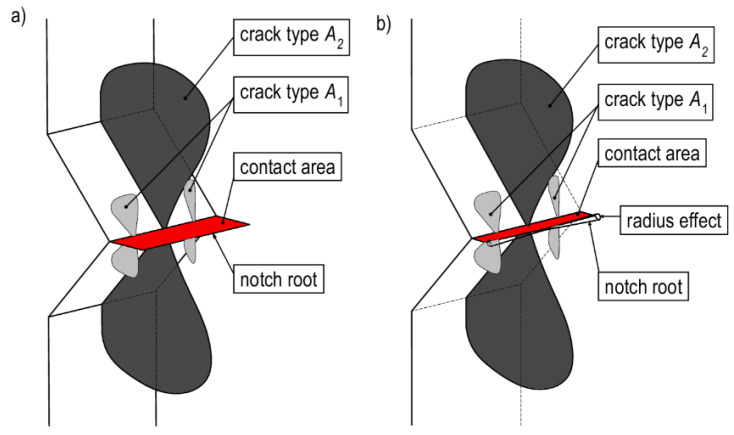
Cracks detected in specimens with (**a**) sharp V-notches and (**b**) rounded V-notches during loading.

The fracture during the loading stage was observed only in specimens with V-notches. The failure was caused by the closing of the notch surfaces. The reason for the fracture initiation was to achieve critical contact stress values, as a result of which A-type cracks were formed, i.e., dragonfly wing (Figure 6). The fracture was initiated from the contact surface edges. The cracks appeared in the middle of the specimen thickness and propagated along the element reaching the end of the observation area (Figure 6, crack type *A*_2_). Cracks of this type also appeared in the vicinity of the specimen’s free wall, which, in turn, propagated at a small distance and were located on planes inclined at an acute angle to the specimen’s symmetry plane (Figure 6, crack type *A*_1_). Fracture initiation moments at all strain rates $\dot{\varepsilon }$ for specimens of thickness *t*_1_ were plotted on the force, i.e., base shortening curves (Figure 4a,b), and the data are presented in Appendix A.

The initiation of the first crack takes place at a certain level of measuring shortening base *u*. For a rate of $\dot{\varepsilon \;}$= 8 × 10^−4^ s^−1^, fracture initiated at an average base shortening level of *u* ≈ 5.75 mm in the sharp notch (the extreme values are rejected) and *u* ≈ 5.88 mm in the rounded notch. An increase in the rate to $\dot{\varepsilon }$ = 4 × 10^−3^ s^−1^ did not significantly affect the fracture initiation moment. A crack appeared at *u* ≈ 5.58 mm in the sharp notch and at *u* ≈ 5.79 mm in the rounded notch. A significant difference was only noticed when the strain rate was 25 times higher, i.e., $\dot{\varepsilon }$ = 2 × 10^−2^ s^−1^. In this case, the first crack was recorded for *u* ≈ 4.57 mm in the sharp notch (the extreme values were rejected) and for *u* ≈ 4.38 mm in the rounded notch. Therefore, only at a rate higher than $\dot{\varepsilon }$ = 4 × 10^−3^ s^−1^, is its influence on the fracture process noticed. This effect disappears with specimens of thickness *t*_2_. Fracture initiation moments at all strain rates $\dot{\varepsilon }$ for specimen thickness *t*_2_ are represented by force—base shortening curves (Figure A1), the data are shown in Appendix A. Regardless of the rate $\dot{\varepsilon }$, the initiation of the first A-type crack always occurred for *u* ≈ 3.2 mm (sharp notch) and *u* ≈ 4.80 mm (rounded notch). A crack in the sharp V-notched specimen had previously initiated due to the full closure of the notch. The larger contact surface resulted in faster achievement of the critical contact stress value. Thus, the effect of rate $\dot{\varepsilon }$ during fracture disappears as the thickness of the specimen increases.

The strain rate $\dot{\varepsilon }$, has not significantly affected the number of A-type cracks appearing, with a *u*_max_ = 7 mm. Cracks of this type appeared symmetrically in two notches and propagated in two directions of the specimen length. For example, for specimens of thickness *t*_2_, there were on average 10–12, 8–10, and 12 cracks, respectively, for the $\dot{\varepsilon }$ equal to 8 × 10^−4^, 4 × 10^−3^, and 2 × 10^−2^ s^−1^.

### 4.2. Fracture During Unloading

**Figure 7:**
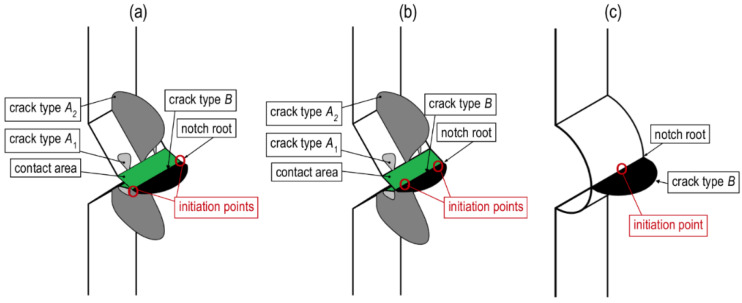
Cracks detected in specimens with (**a**) sharp V-notches; (**b**) rounded V-notches; and (**c**) U-notches during unloading.

During unloading (this stage was carried out ten times faster than loading, $\dot{\varepsilon }$), another type of crack appeared in the notch root, i.e., B-type crack (Figure 7). When the specimen was loaded, close to the notch root (in the symmetry plane of the notches perpendicular to the direction of the loading), negative axial plastic strain and compressive axial normal stress occurred. Their value is limited by yield criterion (including hardening/softening). The unloading stage has an elastic characteristic. As a result, the elastic-plastic axial strain and stress distributions overlap the elastic ones, i.e., tensile axial stress and positive strain. For this reason, tensile normal stress appears in front of the notch during the unloading stage, which causes the fracture initiation in the plane symmetry perpendicular to the load direction. The fracture was initiated by residual stresses, appearing as a result of heterogeneous plastic deformation. The presence of notches caused that locally (near the notch root) the stress exceeded the yield strength while the remaining specimen volume deformed in the elastic range. As a result, tensile residual stresses (responsible for the fracture initiation) were present in the notch root, whereas residual compressive stresses were present in the remaining area, which did not deform plastically.

In V-notched specimens, fracture initiated simultaneously from four points located at the bottom of both notches. Two initiation points were observed in one notch, located on the free surfaces of the element in sharp notches or at some distance from these surfaces in rounded notches. Thus, the rounded bottoms of V-notches significantly influenced the location of the fracture initiation. A different situation was observed in specimens weakened by U-notches, where the fracture initiated from one point located at the notch bottom and the middle of the specimen thickness. It can be seen that the larger the notch root radius, the residual cracks initiate closer to the center of the specimen thickness. There was no effect of rate ${\dot{\varepsilon }}_{2}$ on the location of fracture initiation during unloading.

For the specimen with a thickness of *t*_1_ for a given level of maximum measuring base shortening *u*_max_, together with a reduction in the rate ${\dot{\varepsilon }}_{2}$, lower values of the compressive force at which fracture occurred are observed. For the maximum measuring base shortening level *u*_max_ = 3 mm, a crack in the root of the sharp V-notch initiated at a load of about 22.14% (${\dot{\varepsilon }}_{2}$ = 8 × 10^−3^ s^−1^), 26.18% (${\dot{\varepsilon }}_{2}$ = 4 × 10^−2^ s^−1^), and 30.05% (${\dot{\varepsilon }}_{2}$ = 2 × 10^−1^ s^−1^) of the effective load *F*_eff_. In the presence of rounded V-notches, the fracture initiated at a load of about 28.91% (${\dot{\varepsilon }}_{2}$ = 8 × 10^−3^ s^−1^), 31.48% (${\dot{\varepsilon }}_{2}$ = 4 × 10^−2^ s^−1^), and 33.31% (${\dot{\varepsilon }}_{2}$ = 2 × 10^−1^ s^−1^) of *F*_eff_. Thus, subsequent increases in rate ${\dot{\varepsilon }}_{2}$ resulted in earlier fracture initiation by about 4% (sharp notches) and 2% (rounded notches). It is noted that the presence of rounding accelerates the initiation moment of B-type crack. The smaller contact zone resulting from incomplete notch closing is the reason for a lower maximum compressive force during loading. As a result, in specimens with rounded V-notches, the residual tensile stresses reach a critical value faster. Specimens with U-notches (*t*_1_), during unloading from *u*_max_ = 3 mm, only cracked at ${\dot{\varepsilon }}_{2}$ = 2 × 10^−1^ s^−1^.

This effect disappeared with an increase in the level of the maximum measuring base shortening. For *u*_max_ = 7 mm, the fracture in the sharp notch initiated at a load of about 7.32% (${\dot{\varepsilon }}_{2}$ = 8 × 10^−3^ s^−1^), 6.21% (${\dot{\varepsilon }}_{2}$ = 4 × 10^−2^ s^−1^), and 8.13% (${\dot{\varepsilon }}_{2}$ = 2 × 10^−1^ s^−1^) of *F*_eff_, i.e., at the very end of the process. In the rounded V-notch the, critical load was about 6.73% (${\dot{\varepsilon }}_{2}$ = 8 × 10^−3^ s^−1^), 6.96% (${\dot{\varepsilon }}_{2}$ = 4 × 10^−2^ s^−1^), and 7.71% (${\dot{\varepsilon }}_{2}$ = 2 × 10^−1^ s^−1^) of *F*_eff_. Greater deformation of the specimens caused a delay in the initiation of the residual crack; the tensile residual stresses later reach a critical value. However, similar critical load values expressed as a percentage of *F*_eff_ were observed, regardless of the notch type and rate ${\dot{\varepsilon }}_{2}$. The specimens with U-notches remain in opposition. For *u*_max_ = 7 mm, the fracture in the notch root initiated at a load of about 9.34% (${\dot{\varepsilon }}_{2}$= 8 × 10^−3^ s^−1^) and 2.79% (${\dot{\varepsilon }}_{2}$ = 4 × 10^−2^ s^−1^) *F*_eff_. At a load rate of ${\dot{\varepsilon }}_{}$ = 2 × 10^−1^ s^−1^, the specimens were buckled, and therefore rejected from the analysis.

The rate effect is more pronounced for thicker specimens (*t*_2_). The greatest differences in the values of the compressive force at which the residual crack initiation took place were observed for the lowest values of maximum base shortening level. For *u*_max_ = 3 mm, fracture in the sharp notch initiated at a load of about 20.93% (${\dot{\varepsilon }}_{2}$ = 8 × 10^−3^ s^−1^), 31.34% (${\dot{\varepsilon }}_{2}\;$= 4 × 10^−2^ s^−1^), and 29.54% (${\dot{\varepsilon }}_{2}$ = 2 × 10^−1^ s^−1^) of *F*_eff_. In the case of rounded V-notches, the fracture initiation was recorded at a load of about 31.50% (${\dot{\varepsilon }}_{2}$ = 8 × 10^−3^ s^−1^), 37.76% (${\dot{\varepsilon }}_{2}$= 4 × 10^−2^ s^−1^), and 52.61% (${\dot{\varepsilon }}_{2}$ = 2 × 10^−1^ s^−1^) of *F*_eff_. A significant acceleration of the fracture initiation was observed with an increase in the unloading rate, especially for specimens with rounded notches, even by up to 20%. In components with sharp notches at higher unloading rates, fracture initiated at a similar load level expressed as a percentage of *F*_eff_. Specimens with U-notches do not break at this level of maximum base shortening. For *u*_max_ = 7 mm, the fracture in notch root initiated at loads of about 5.78% (${\dot{\varepsilon }}_{2}$ = 8 × 10^−3^ s^−1^), 9.95% (${\dot{\varepsilon }}_{2}$ = 4 × 10^−2^ s^−1^), and 11.10% (${\dot{\varepsilon }}_{2}$ = 2 × 10^−1^ s^−1^) of *F*_eff_. Further rate ${\dot{\varepsilon }}_{2}$ increases resulted in earlier fracture initiation. The rounding of the V-notch, in this case, resulted in a delayed the fracture initiation, which took place at loads of about 4.72% (${\dot{\varepsilon }}_{2}$ = 8 × 10^−3^ s^−1^), 5.85% (${\dot{\varepsilon }}_{2}$ = 4 × 10^−2^ s^−1^), and 6.53% (${\dot{\varepsilon }}_{2}$ = 2 × 10^−^1 s^−1^) of *F*_eff_. As the rate ${\dot{\varepsilon }}_{2}$ increases, with sharp notched specimens occurring more quickly, in contrast to a maximum base shortening of *u*_max_ = 3 mm. These differences result from different contact area sizes, as described in Section 4. It has been shown that at the same level of measuring base shortening a different stage of notch closure can be observed, so the contact areas are different. Sharp notch surfaces are in contact earlier than in rounded ones. The situation is analyzed to the level of base shortening exceeding the moment when the critical contact area is reached (step on compression curves).

In specimens with rounded V-notches from this moment, the contact area increases faster. This results in a larger contact area (higher compressive force values) as compared with specimens with sharp notches. Thus, the tensile residual stresses reach a critical value later, which determines the fracture initiation moment. Specimens with rounded V-notches at *u*_max_ = 7 mm were destroyed when the load reached about 1.30% (${\dot{\varepsilon }}_{2}$ = 8 × 10^−3^ s^−1^), 4.67% (${\dot{\varepsilon }}_{2}$ = 4 × 10^−2^ s^−1^), and 4.59% (${\dot{\varepsilon }}_{2}$ = 2 × 10^−1^ s^−1^) of *F*_eff_. It can be noted that the larger the rounding of the notch root radius, the higher the unloading rate, and the fracture was initiated later. A contact zone is not observed in samples with U-notches. The lack of a strong stress concentration in the notch root led to an enlarged plastic deformation zone and the tensile residual stresses later reached a critical value.

**Figure A2:**
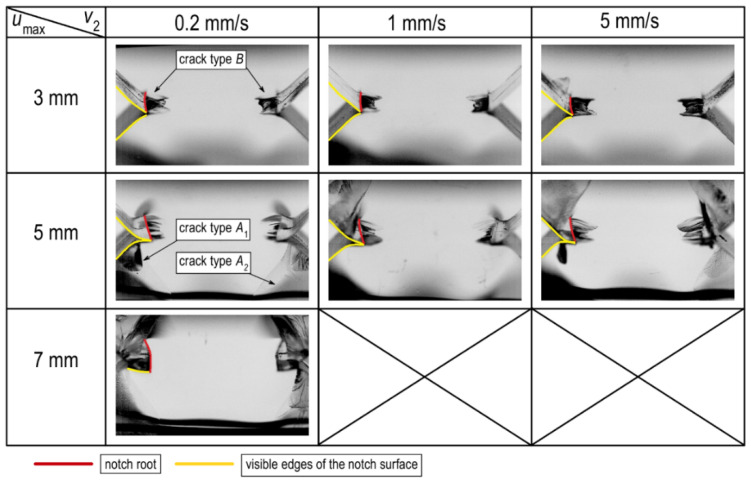
Deformation in the sharp V-notched specimens (*t*_2_) after the unloading for *u*_max_ = 3, 5, and 7 mm and ${\dot{\varepsilon }}_{2}$ 8 × 10^−3^, 4 × 10^−2^, and 2 × 10^−1^ s^−1^_._

**Figure A3:**
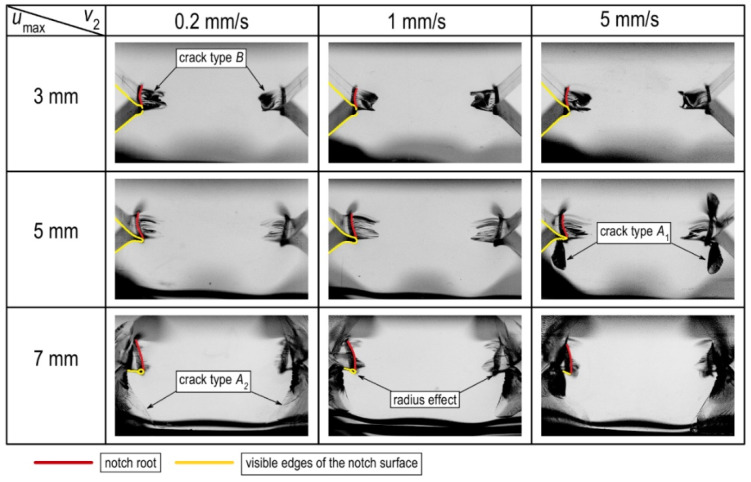
Deformation in the rounded V-notched specimens (*t*_2_) after the unloading for *u*_max_ = 3, 5, and 7 mm and ${\dot{\varepsilon }}_{2}$ 8 × 10^−3^, 4 × 10^−2^, and 2 × 10^−1^ s^−1^_._

**Figure A4:**
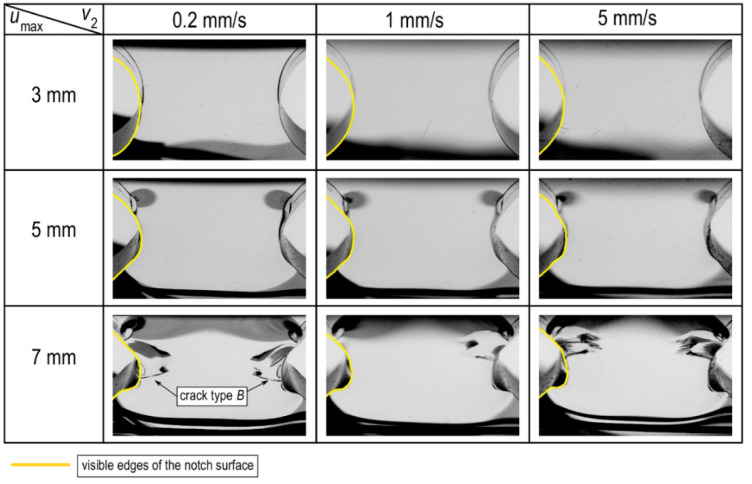
Deformation in the U-notched specimens (*t*_2_) after the unloading for *u*_max_ = 3, 5, and 7 mm and ${\dot{\varepsilon }}_{2}$ 8 × 10^−3^, 4 × 10^−2^, and 2 × 10^−1^ s^−1^.

The higher the level of maximum base shortening *u*_max_, the larger the plastic deformation zone and a larger contact area is observed. The higher material softening results in the occurrence of tensile residual stresses over a larger area around the notch root and contact area. Increasing the unloading speed, results in an increase in yield strength, and thus earlier reaching of the critical value of the tensile residual stresses, with a higher percentage of *F*_eff_. Regardless of the rate ${\dot{\varepsilon }}_{2}$, the fracture in all the specimens initiated at similar levels of *u*. It should be noted that the loading and unloading rate did not affect the evolution of B-type cracks. Photos of specimens with thickness *t*_2_ after the unloading are shown in Figure A2, Figure A3 and Figure A4. In order to make the interpretation of the graphs easier, on the one side of the specimen, the notch bottom (red) and closer edges of the notch surface (yellow) are marked. Additionally, selected A- and B-type cracks were indicated. A comparison of the shape of the crack (in the notch root) for the same levels of measuring base shortening but different strain rates showed that there no noticeable variations. The fracture surfaces are similar in shape and size. For a rate of 8 × 10^−4^ s^−1^ to 8 × 10^−3^ s^−1^, the processes of residual crack development for individual notch types are described in detail in [26].

## 5. Conclusions

This paper presents the results of experimental studies on the uniaxial compressive load rate effect (including unloading) on the fracture in notched elements made of polymethyl methacrylate (PMMA). Different shapes of V-notches (sharp and rounded) and U-notches, as well as two different specimen thicknesses, were taken into account. The study showed that the type and size of the load, and also the time of its rise, were important in the engineering analysis of PMMA elements with notches.

Depending on the notch type, the compression curves with unloading presented different characteristics. The higher the strain rate, the higher the compressive force values were recorded. The specimens with V-type notches showed a continuous increase in the compressive force values along with an increased level of the measuring base shortening. In the case of U-notches, a decrease in the load value was visible. Thus, the larger the notch root radius, the lower the concentration of stress and strain and the more the three stages that are characteristics of amorphous polymers were visible, i.e., viscoelastic deformation, softening, and hardening. For all notch types and levels of the measuring base shortening *u*, at the end of the unloading, the same permanent base shortening was observed (regardless of the applied strain rate).

The fracture initiation moments, which correspond to specific critical load values, were indicated. The first type of crack was observed during unloading. A-type crack, i.e., dragonfly wing, appeared only in specimens with V-type notches due to the critical value of contact stress. The fracture was initiated from the contact surface edges. The higher the strain rate, the higher the compressive force causing the initiation of fracture. Regardless of the strain rate, in thicker specimens, these cracks were indicated earlier in the presence of sharp V-notches (*u* ≈ 3.2 mm) than in rounded ones (*u* ≈ 4.8 mm). Studies have shown that the process of closing the sharp notch starts much earlier that with rounded notches. This is equivalent to the fact that, for a given level of the measuring base shortening, a larger contact area was recorded in specimens with sharp notches, which explains the earlier fracture initiation. However, the strain rate does not significantly affect the location and shape of cracks. Another type of crack appeared during the unloading stage as a result of tensile residual stress. The fracture initiated in all types of notches, with different values of compression force. The shape of the notch strongly influences the location of fracture initiation. These locations change from free surfaces (sharp V-notches) to the middle of the specimen thickness (U-notches). The crack B-type initiation moment is influenced by the course of the loading stage. The smaller the contact area resulting from incomplete notch closure results in a lower maximum compressive force during loading. As a result, in specimens with rounded V-notches, the residual tensile stress reaches a critical value more quickly. No contact area is observed in specimens with U-notches. The lack of a strong stress concentration in the notch root led to an enlarged plastic deformation zone and the tensile residual stresses later reached a critical value. It was shown that its influence on the fracture process is noticed only at a strain rate higher than $\dot{\varepsilon }$ = 4 × 10^−3^ s^−1^. This rate effect disappears as the thickness of the specimen increases.

The presented test results can be the basis for numerical modeling of stress and strain fields in notched specimens using the finite element method, to formulate the fracture criterion of the notched element under compressive loading. Such studies are planned in the next stage by the authors of this paper.

## Figures and Tables

**Table 1:** PHANTOM camera’s parameters.

Strain Rate Loading	Strain Rate Unloading	Unloading Displacement	Recording Rate	Exposure Time
(s^−1^)	(s^−1^)	(mm)	(frame/s)	(µs)
8 × 10^−4^	8 × 10^−3^	3	2200	440
-	-	5	1500	660
-	-	7	1080	920
4 × 10^−3^	4 × 10^−2^	3	7980	120
-	-	5	6000	160
-	-	7	4980	200
2 × 10^−2^	2 × 10^−1^	3	15,000	66
-	-	5	-	-
-	-	7	-	-

## Nomenclature

*A*_nom_	nominal cross-sectional area of the specimen measured at the bottom of the notch
*A*_real_	real cross-sectional area of the specimen measured at the bottom of the notch
*E*	Young’s modulus [MPa]
*F*_max_	maximum compressive force (kN)
*F*_eff_	effective load (*F*_eff_ = *ξ* · *F*_max_) (kN)
*u*	measuring base shortening (mm)
*u*_max_	maximum level of measuring base shortening (mm)
$\dot{u}$	measuring base shortening rate during loading (mm/s)
${\dot{u}}_{2}$	measuring base shortening rate during unloading (mm/s)
*t*_1_, *t*_2_	specimen thicknesses (mm]
*R*	notch radius (mm)
*R*_c_	Compressive strength (MPa)
*R*_m_	Tensile strength (MPa)
*R*_u_	Failure stress (MPa)
*R*_0.2_	Yield stress (MPa)
*R*_0.05_	Elastic limit (MPa)
*v*	Poisson’s ratio
*ξ*	correction factor (*ξ* = *A*_nom_/*A*_real_)
$\dot{\varepsilon }$	mean strain rate during loading (s^−1^)
${\dot{\varepsilon }}_{2}$	mean strain rate during unloading (s^−1^)

## Appendix A

**Table A1:** PMMA’s properties at a displacement rate of 0.02 mm/s [26].

Material Tensile Property	Value	Material Compressive Property	Value
Young’s modulus, *E* (MPa)	3311	Young’s modulus, *E* (MPa)	3702
Poisson’s ratio, *v*	0.38	Poisson’s ratio, *v*	0.37
Yield stress, *R*_0.2_ (MPa)	48	Yield stress, *R*_0.2_ (MPa)	71
Elastic limit, *R*_0.05_ (MPa)	34	Elastic limit, *R*_0.05_ (MPa)	53
Tensile strength, *R*_m_ (MPa)	72	Compressive strength, *R*_c_ (MPa)	116
Failure stress, *R*_u_ (MPa)	71		

**Table A2:** Fracture load and displacement for the samples with sharp V-notches.

Loading Rate (mm/s)	Unloading Rate (mm/s)	Nominal Sample Thickness (mm)	Loading Displacement (mm)	Sample	Cross Section (mm × mm)	Max. Load *F*_max_ (kN)	Effective Load *F* * (kN)	Crack Type *A* Initiation (Loading)		Crack Type *B* Initiation (Unloading)	
								Displacement (mm)	Load (kN)	Displacement (mm)	Load (kN)
0.02	0.2	9.70	3	V10-1	9.64 × 19.18	24.45	25.65	-	-	1.85	4.76
				V10-2	9.64 × 19.40	25.07	26.01	-	-	1.98	6.21
			5	V10-3	9.67 × 19.19	25.58	26.74	-	-	3.85	4.09
				V10-4	9.70 × 19.34	26.19	27.08	4.48	25.82	3.83	4.10
			7	V10-5	9.67 × 19.20	27.61	28.85	5.95	26.42	5.66	2.27
				V10-6	9.71 × 19.11	27.96	29.23	5.54	26.33	5.57	1.80
		14.50	3	V15-1	14.66 × 19.19	40.86	42.12	-	-	1.87	9.79
				V15-2	14.64 × 19.12	41.10	42.58	-	-	1.69	7.37
			5	V15-3	14.70 × 19.02	41.23	42.76	3.28	40.79	3.54	3.82
				V15-4	14.56 × 19.13	40.52	42.19	3.42	40.09	3.57	3.56
			7	V15-5	14.41 × 19.29	42.61	44.45	3.58	39.67	5.44	1.55
				V15-6	14.43 × 19.25	42.70	44.58	3.16	39.41	5.64	3.38
0.1	1	9.70	3	V10-7	9.88 × 19.24	29.64	30.25	-	-	2.03	8.75
				V10-8	9.64 × 19.18	27.44	28.79	-	-	1.87	6.22
			5	V10-9	9.71 × 19.25	29.95	31.08	-	-	3.71	4.00
				V10-10	9.87 × 19.18	29.14	29.86	-	-	3.81	4.89
			7	V10-11	9.62 × 19.26	31.52	33.00	5.28	29.79	5.47	1.75
				V10-12	9.67 × 19.32	30.48	31.65	5.88	29.25	5.54	2.10
		14.50	3	V15-7	14.67 × 19.00	43.99	45.77	-	-	2.05	14.83
				V15-8	14.37 × 19.26	43.76	45.85	-	-	1.95	12.68
			5	V15-9	14.43 × 19.22	44.74	46.78	3.32	44.58	3.52	4.70
				V15-10	14.39 × 19.28	46.22	48.31	3.20	46.19	3.51	4.35
			7	V15-11	14.42 × 19.20	*	*	3.11	45.87	-	-
				V15-12	14.40 × 19.20	*	*	*	*	-	-
0.5	5	9.70	3	V10-13	9.63 × 19.24	31.39	32.87	-	-	1.91	8.15
				V10-14	9.62 × 19.16	31.19	32.83	-	-	2.09	10.65
			5	V10-15	9.78 × 19.16	32.41	33.55	4.60	32.34	3.91	7.08
				V10-16	9.64 × 19.41	32.05	33.23	4.57	32.07	3.87	6.38
			7	V10-17	9.66 × 19.27	33.71	35.13	6.60	33.64	5.57	2.77
				V10-18	9.64 × 19.31	33.33	34.74	3.54	31.31	5.59	2.68
		14.50	3	V15-13	14.49 × 19.00	50.72	53.43	-	-	1.89	14.27
				V15-14	14.48 × 19.00	51.33	54.11	-	-	1.94	15.87
			5	V15-15	14.40 × 19.20	51.07	53.57	2.99	50.94	3.49	5.48
				V15-16	14.50 × 19.10	51.67	54.10	2.98	51.19	3.54	5.92
			7	V15-17	14.39 × 19.08	51.63	54.53	3.14	50.51	*	*
				V15-18	14.37 × 19.13	*	*	*	*	*	*

* final failure before unloading.

**Table A3:** Fracture load and displacement for the samples with rounded V-notches.

Loading Rate (mm/s)	Unloading Rate (mm/s)	Nominal Sample Thickness (mm)	Loading Displacement (mm)	Sample	Cross- Section (mm × mm)	Max. Load *F*_max_ (kN)	Effective Load *F* * (kN)	Crack Type *A* Initiation (Loading)		Crack Type *B* Initiation (Unloading)	
								Displacement (mm)	Load (kN)	Displacement (mm)	Load (kN)
0.02	0.2	9.70	3	VR10-1	9.64 × 20.01	24.60	23.99	-	-	2.08	7.22
				VR10-2	9.77 × 20.02	24.90	24.70	-	-	2.06	7.09
			5	VR10-3	9.71 × 20.11	25.72	25.55	-	-	3.66	2.51
				VR10-4	9.77 × 20.12	25.64	25.30	-	-	3.80	3.33
			7	VR10-5	9.77 × 20.09	28.99	28.65	5.88	27.56	5.73	2.89
				VR10-6	9.62 × 20.02	28.44	28.65	5.89	27.05	5.38	0.99
		14.50	3	VR15-1	14.35 × 20.07	40.37	40.65	-	-	2.05	12.79
				VR15-2	14.48 × 19.87	40.19	40.51	-	-	2.04	12.59
			5	VR15-3	14.41 × 20.03	40.49	40.68	-	-	3.64	5.05
				VR15-4	14.45 × 20.02	40.51	40.61	-	-	3.64	5.14
			7	VR15-5	14.51 × 20.00	43.12	43.09	5.01	40.45	5.46	1.73
				VR15-6	14.44 × 20.07	43.04	43.07	5.06	40.31	5.53	2.34
0.1	1	9.70	3	VR10-7	9.89 × 20.12	29.47	28.73	-	-	2.09	9.48
				VR10-8	9.77 × 20.04	28.68	28.42	-	-	2.07	8.83
			5	VR10-9	9.64 × 20.15	29.54	29.50	-	-	3.82	5.18
				VR10-10	9.89 × 20.08	29.71	29.02	-	-	3.67	3.40
			7	VR10-11	9.70 × 20.19	31.37	31.07	-	-	5.62	2.72
				VR10-12	9.64 × 20.08	31.04	31.11	5.79	29.67	5.50	1.63
		14.50	3	VR15-7	14.46 × 19.95	44.48	44.71	-	-	2.12	16.40
				VR15-8	14.51 × 20.02	45.40	45.32	-	-	2.16	17.54
			5	VR15-9	14.55 × 19.94	45.45	45.43	-	-	**	**
				VR15-10	14.52 × 20.01	45.71	45.62	-	-	3.66	6.66
			7	VR15-11	14.51 × 20.06	47.63	47.45	5.11	45.31	5.52	3.18
				VR15-12	14.44 × 20.12	47.69	47.60	4.85	45.31	5.45	2.40
0.5	5	9.70	3	VR10-13	9.62 × 19.98	31.46	31.75	-	-	2.03	9.84
				VR10-14	9.88 × 20.00	32.65	32.06	-	-	2.11	11.53
			5	VR10-15	9.62 × 20.09	32.38	32.50	4.46	32.46	3.78	5.90
				VR10-16	9.63 × 20.12	32.10	32.14	4.58	31.11	3.80	5.62
			7	VR10-17	9.78 × 20.18	34.90	34.31	4.26	33.13	5.56	2.23
				VR10-18	9.63 × 20.04	33.75	33.93	4.20	31.82	5.59	3.05
		14.50	3	VR15-13	14.60 × 20.01	52.71	52.32	-	-	2.37	27.53
				VR15-14	14.59 × 20.00	53.22	52.89	-	-	2.38	28.20
			5	VR15-15	14.58 × 20.08	53.37	52.87	4.49	53.14	3.93	13.52
				VR15-16	14.50 × 20.08	52.49	52.28	4.56	52.40	3.76	10.39
			7	VR15-17	14.71 × 19.98	54.58	53.85	4.99	53.20	5.48	3.11
				VR15-18	14.70 × 20.05	*	*	4.35	52.97	5.54	4.03

* final failure before unloading, ** no data.

**Table A4:** Fracture load and displacement for the samples with U-notches.

Loading Rate (mm/s)	Unloading Rate (mm/s)	Nominal Sample Thickness (mm)	Loading Displacement (mm)	Sample	Cross-Section (mm × mm)	Max. Load Fmax (kN)	Effective Load F * (kN)	Crack Type B Initiation (Unloading)	
								Displacement (mm)	Load (kN)
0.02	0.2	9.70	3	U10-1	9.71 × 20.10	24.57	24.42	-	-
				U10-2	9.66 × 20.17	24.59	24.48	-	-
			5	U10-3	9.64 × 20.19	24.92	24.84	3.72	3.14
				U10-4	9.89 × 20.28	24.50	23.70	-	-
			7	U10-5	9.71 × 20.11	26.58	26.41	5.58	2.03
				U10-6	9.88 × 20.15	27.10	26.41	5.68	2.99
		14.50	3	U15-1	14.54 × 19.95	38.83	38.82	-	-
				U15-2	14.62 × 19.94	38.41	38.21	-	-
			5	U15-3	14.63 × 19.83	38.63	38.61	*	
				U15-4	14.74 × 19.70	38.50	38.45	*	
			7	U15-5	14.43 × 20.08	39.89	39.92	5.37	1.04
				U15-6	14.48 × 20.07	40.05	39.97	5.41	1.55
0.1	1	9.70	3	U10-7	9.77 × 20.18	27.57	27.13	-	-
				U10-8	9.67 × 20.10	27.31	27.26	-	-
			5	U10-9	9.70 × 20.12	26.50	26.34	3.50	1.61
				U10-10	9.68 × 20.13	26.72	26.60	-	-
			7	U10-11	9.71 × 20.14	27.83	27.61	5.59	1.84
				U10-12	9.71 × 20.22	30.03	29.67	5.53	1.69
		14.50	3	U15-7	14.57 × 19.92	42.73	42.70	-	-
				U15-8	14.55 × 19.97	42.68	42.60	-	-
			5	U15-9	14.67 × 19.89	43.39	43.12	*	
				U15-10	14.66 × 19.88	43.19	42.98	*	
			7	U15-11	14.57 × 20.03	42.99	42.72	5.37	2.02
				U15-12	14.77 × 19.93	43.54	42.89	5.39	2.26
0.5	5	9.70	3	U10-13	9.77 × 20.33	32.17	31.42	1.94	9.62
				U10-14	9.87 × 20.23	32.21	31.30	-	-
			5	U10-15	9.88 × 20.24	30.73	29.81	-	-
				U10-16	9.71 × 20.25	30.03	29.63	3.36	1.04
			7	U10-17	9.67 × 20.25	30.87	30.58	**	
				U10-18	9.67 × 20.23	31.97	31.70	**	
		14.50	3	U15-13	14.43 × 19.99	47.87	48.13	-	-
				U15-14	14.42 × 19.95	48.16	48.55	-	-
			5	U15-15	14.35 × 20.06	48.43	48.79	*	
				U15-16	14.41 × 20.02	48.03	48.28	*	
			7	U15-17	14.48 × 19.98	48.64	48.76	5.34	2.35
				U15-18	14.42 × 20.07	47.89	47.99	5.51	4.65

* crack after the test (outside the handles); ** buckling.
